# MK-0677, a Ghrelin Agonist, Alleviates Amyloid Beta-Related Pathology in 5XFAD Mice, an Animal Model of Alzheimer’s Disease

**DOI:** 10.3390/ijms19061800

**Published:** 2018-06-18

**Authors:** Yu-on Jeong, Soo Jung Shin, Jun Yong Park, Bo Kyeong Ku, Ji Soo Song, Jwa-Jin Kim, Seong Gak Jeon, Sang Min Lee, Minho Moon

**Affiliations:** 1 Department of Biochemistry, College of Medicine, Konyang University, Daejeon 35365, Korea; yuon918@naver.com (Y.-o.J.); tlstnzz83@gmail.com (S.J.S.); kid1tony@naver.com (J.Y.P.); qhrud21410@gmail.com (B.K.K.); songkuku@naver.com (J.S.S.); 2 Department of Biomedical Science, Jungwon University, Geosan, Chungbuk 28024, Korea; kjj1021@naver.com; 3 Department of Nephrology, School of Medicine, Chungnam National University, Daejeon 35015, Korea; 4 Department of Psychiatry, Konyang University College of Medicine, Konyang University Hospital, Daejeon 35365, Korea; 5 Myunggok Medical Research Institute, Konyang University, Daejeon 35365, Korea

**Keywords:** Alzheimer disease, ghrelin, MK-0677, amyloid beta, 5XFAD mice, Ibutamoren

## Abstract

Alzheimer’s disease (AD) is a progressive neurodegenerative disorder characterized by cognitive deficits, neuroinflammation, and neuronal death. The primary pathogenic cause is believed to be the accumulation of pathogenic amyloid beta (Aβ) assemblies in the brain. Ghrelin, which is a peptide hormone predominantly secreted from the stomach, is an endogenous ligand for the growth hormone secretagogue-receptor type 1a (GHS-R1a). MK-0677 is a ghrelin agonist that potently stimulates the GHS-R1a ghrelin receptor. Interestingly, previous studies have shown that ghrelin improves cognitive impairments and attenuates neuronal death and neuroinflammation in several neurological disorders. However, it is unknown whether MK-0677 can affect Aβ accumulation or Aβ-mediated pathology in the brains of patients with AD. Therefore, we examined the effects of MK-0677 administration on AD-related pathology in 5XFAD mice, an Aβ-overexpressing transgenic mouse model of AD. MK-0677 was intraperitoneally administered to three-month-old 5XFAD mice. To visualize Aβ accumulation, neuroinflammation, and neurodegeneration, thioflavin-S staining and immunostaining with antibodies against Aβ (4G8), ionized calcium-binding adaptor molecule 1 (Iba-1), glial fibrillary acidic protein (GFAP), neuronal nuclear antigen (NeuN), and synaptophysin were conducted in the neocortex of 5XFAD and wild-type mice, and to evaluate changes of phosphorylated cyclic adenosine monophosphate (cAMP) response element binding protein (pCREB) levels, immunostaining with antibody against pCREB was performed in dentate gyrus of the hippocampus of 5XFAD and wild-type mice. The histological analyses indicated that MK-0677-treated 5XFAD mice showed reduced Aβ deposition, gliosis, and neuronal and synaptic loss in the deep cortical layers, and inhibited the decrement of pCREB levels in dentate gyrus of the hippocampus compared to vehicle-treated 5XFAD mice. Our results showed that activation of the ghrelin receptor with MK-0677 inhibited the Aβ burden, neuroinflammation, and neurodegeneration, which suggested that MK-0677 might have potential as a treatment of the early phase of AD.

## 1. Introduction

Alzheimer’s disease (AD) is the most preeminent type of dementia. The number of Americans affected by AD is increasing exponentially and is expected to reach 13.8 million by 2050 from 5.4 million in 2016 [1,2]. AD results in various symptoms, such as memory impairments, language disturbances, and psychiatric problems [3]. The more AD progresses, the more daily functioning decreases and neuropsychiatric symptoms increase. According to the amyloid hypothesis, which is the most well-developed of the hypotheses of AD pathogenesis, deposits of the amyloid beta (Aβ) peptide are considered the major cause of the development of AD [4]. In addition, Aβ directly causes neurodegeneration, microgliosis, astrocytosis, neurofibrillary tangle deposition, and memory loss [4]. Therefore, targeting Aβ aggregation and Aβ-related pathologic changes has been suggested as a potential strategy for preventing AD pathogenesis.

Ghrelin, which is a 28-amino-acid peptide hormone, is mostly released from gastric enteroendocrine cells. The release of ghrelin can be caused by hunger [5]. In addition, des-acyl ghrelin, an inactive form of ghrelin, acts on the growth hormone secretagogue receptor type 1a (GHS-R1a) when it is transformed into acylated ghrelin by ghrelin o-acyltransferase (GOAT) [6]. Ghrelin is expressed in various cells and organs, such as the stomach, testis, ovary, kidney, and small intestine [7,8,9,10,11,12,13,14]. Several studies have suggested that ghrelin-containing neurons are extensively expressed in the central nervous system (CNS) [15,16,17]. In addition, accumulating evidence for the role of the ghrelin system in cognitive functions has indicated that they are strongly correlated [18,19]. One study reported that the mRNA levels of ghrelin, GOAT, and GHS-R1a were decreased in the temporal lobe of patients with AD [20]. A clinical study has revealed that male patients who were newly diagnosed with AD had a decreased area under the curve for ghrelin levels, while female patients did not show any alterations [21]. Therefore, the cognitive impairments in patients with AD have been suggested to be associated with the altered ghrelin system.

Ghrelin has various effects on physiological functions, such as appetite regulation [22], adiposity [23], glucose metabolism [24], and energy homeostasis in the hypothalamus [25,26,27]. Moreover, studies have revealed an association between ghrelin and neural function. Previous in vitro studies have demonstrated that ghrelin treatment enhances the synaptic density of dissociated cortical neurons with decreased synapses induced by hypoxia [28], attenuates oxygen/glucose deprivation-induced apoptosis in hypothalamic neurons [29], increases the proliferation of hippocampal neural stem cells via multiple signaling pathways [30], prevents apoptosis signal-regulating kinase 1-mediated apoptosis of rat pheochromocytoma (PC12) cells by increasing heat-shock protein 70 levels [31], and inhibits the increment of inflammatory cytokines induced by fibrillar Aβ in mice microglial cells [32]. Consistent with the in vitro studies, ghrelin has protective effects on the CNS in vivo. Ghrelin administration ameliorates the neuronal damage of hippocampal neurons in ischemia/reperfusion-injured rats [33] and rats with pilocarpine-induced seizures [34], and inhibits neuronal loss in the substantia nigra pars compacta (SNpc) in a 1-methyl-4-phenyl-1,2,5,6 tetrahydropyridine (MPTP)-induced mouse model of Parkinson’s disease [35]. Moreover, ghrelin administration improves spatial learning and memory in animal models. The cognitive improvements induced by ghrelin are mediated by an increased spine synapse density in the CA1 of the hippocampus of rats [36] and the stimulation of adult hippocampal neurogenesis in the subgranular zone of the hippocampus of mice [37]. In accordance with the effects of ghrelin in AD, studies using animal AD models have suggested that ghrelin alleviates Aβ-induced synaptic degeneration, microgliosis, impaired adult hippocampal neurogenesis, and cognitive deficits [38,39]. Taken together, these results surprisingly indicate that ghrelin has beneficial properties against neurotoxicity and pathologic changes that are similar to Aβ-induced pathogenesis.

MK-0677, which is also known as ibutamoren mesylate or L-163,191, is a non-peptide ghrelin agonist with higher efficacy compared to ghrelin when it is bound to GHS-R1a [40,41,42]. The half-life of MK-0677 is 6 h, which is approximately 12 times longer than ghrelin’s 30 min [43,44]. In addition, MK-0677 increases the levels of growth hormone and insulin-like growth factor-1 [45]. Therefore, the properties of MK-0677 can be used to stimulate the ghrelin receptor, which could be effective for enhancing degenerated cognitive functions. Nevertheless, the effects of MK-0677 on AD pathology in animal models have not yet been reported.

To the finest of our knowledge, only a few studies have investigated a ghrelin agonist (LY444711) or ghrelin antagonist ([d-Lys3]-GHRP-6) in animal models of AD. Moreover, there were considerable discrepancies in the effects of ghrelin agonists on Aβ load in the previous studies. The long-term administration of LY444711 significantly increases cognitive performance and lowers Aβ levels in the dentate gyrus in an AD mouse model [46]. However, another study reported that the long-term treatment of LY444711 did not change Aβ levels in the dentate gyrus, stratum oriens, and olfactory bulb, while cognitive functions were improved in the AD mouse model [47]. Moreover, [d-Lys3] GHRP-6, a ghrelin antagonist, reduced Aβ levels in the hippocampus in rats with monosodium glutamate-induced obesity [48]. In a randomized clinical trial that did not examine Aβ levels, the administration of MK-0677 did not improve cognitive function in patients with mild to moderate AD [49]. These disparities with the results of the studies using ghrelin agonists or antagonists confuse the development of strategies for treating AD. Therefore, to clarify the effects of MK-0677 on Aβ and Aβ-induced pathogenesis, we aimed to test the efficacy of the ghrelin agonist MK-0677 on AD pathology, including Aβ accumulation, neurodegeneration, and neuroinflammation in mice at the early phase of AD through histological quantification analyses. In addition, we purposed to evaluate the role of MK-0677 in levels of phosphorylation of the cyclic adenosine monophosphate (cAMP) response element binding protein (pCREB), which is responsible for cognitive functioning and neuronal survival [50,51], in dentate gyrus of the hippocampus. Here, we report that the administration of MK-0677 ameliorates Aβ accumulation, neuronal/synaptic loss, microgliosis, and astrogliosis, and inhibits the decrement of the phosphorylation of CREB in the hippocampus without altering significant appetite in three-month-old 5XFAD mice.

## 2. Results

### 2.1. Ghrelin Agonist Treatment Affected the Food Intake and Body Weight of the Healthy Animals

**Figure 1:**
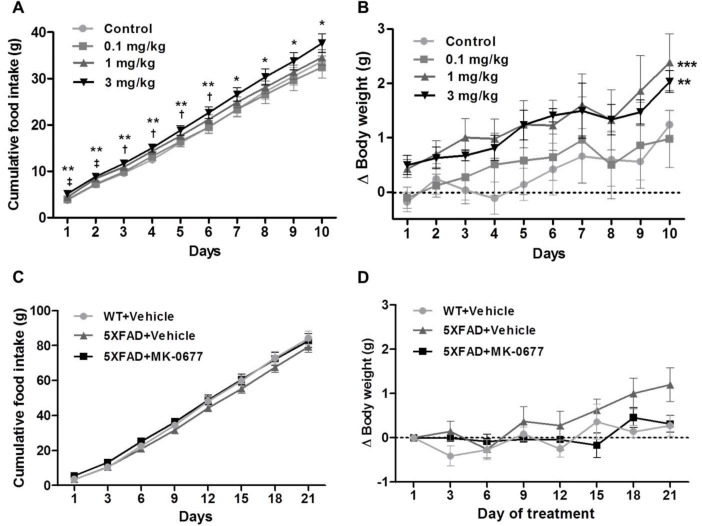
Cumulative food intake and change in body weight after the administration of MK-0677 in C57BL/6 mice and 5XFAD mice. MK-0677 was administered daily to C57BL/6 mice (*n* = 5) at doses of 0.1, 1, and 3 mg/kg for 10 days and to 5XFAD mice at doses of 5 mg/kg for three weeks. The group injected with MK-0677 exhibited a significant increase in cumulative food intake compared with the control group. Compared with the control group, the significant difference indicators are as follows: 0.1 mg/kg group (^†^ *p* < 0.05), 1 mg/kg group (^‡^ *p* < 0.001), and 3 mg/kg group (* *p* < 0.05 and ** *p* < 0.01) (**A**). The body weight changes for 10 days were also significantly increased in the group receiving MK-0677 (** *p* < 0.01 and *** *p* < 0.001 indicate significant differences compared to control group) (**B**). MK-0677-treated 5XFAD mice (*n* = 8) showed tendency to the increase of cumulative food intake induced compared with vehicle-treated 5XFAD mice (*n* = 8) (**C**). The body weight changes among wild-type mice (*n* = 8), vehicle- and, MK-0677-treated 5XFAD mice were not significantly different (**D**).

The ghrelin agonist MK-0677 has acute orexigenic effects in rodents [52]. To establish the optimal doses of MK-0677 that induce biological responses, we injected eight-week-old C57BL/6 mice with 0.1, 1, and 3 mg/kg of MK-0677 cumulatively for 10 days, as described previously [52]. The intraperitoneal administration of 1 and 3 mg/kg of MK-0677 increased food intake compared to that in the vehicle-administered group (*n* = 5; Figure 1A). In particular, the 3 mg dose significantly increased cumulative food intake during the 10-day administration period. In addition, the body weight of the mice administered MK-0677 at 1 mg/kg (92.6%) and 3 mg/kg (64.1%) was significantly higher than that of the vehicle-treated animals at 10 days (Figure 1B). The administration of MK-0677 to the three-month-old 5XFAD mice (*n* = 8) showed a tendency for increased cumulative food intake compared to that in the vehicle-treated 5XFAD mice (Figure 1C). However, the changes in body weight after treatment with MK-0677 did not differ significantly (Figure 1D).

### 2.2. Ghrelin Agonist Treatment Significantly Reduces Aβ Accumulation in the Brains of 5XFAD Mice

**Figure 2:**
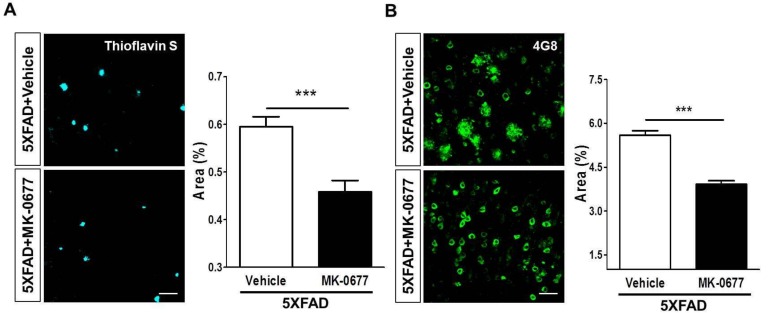
MK-0677 treatment significantly reduced Aβ plaques in the deep cortical layers of 5XFAD mice. The burden of Aβ was estimated by thoflavin-S staining and immunohistochemical staining for the 4G8 antibody. 5XFAD mice treated with MK-0677 (*n* = 6) showed a decreased positive area (%) in both thioflavin-S (**A**) and 4G8 (**B**)-stained brains, compared with vehicle-treated 5XFAD mice (*n* = 7). *** *p* < 0.001 indicates significant differences between the groups. Scale bar = 50 μm.

To test if activation of the ghrelin receptor affected β-amyloidosis, we performed thioflavin-S staining in the frontal cortex of 5XFAD mice. The quantitative analysis demonstrated that the Aβ burden in the deep cortical layers was significantly decreased to 23% after the MK-0677 injections in the 5XFAD mice compared to the vehicle-administered 5XFAD mice (Figure 2A). In addition, the 4G8 immunoreactivity in layer V of the frontal cortex showed that MK-0677 administration significantly decreased the 4G8-positive areas to 30% compared with that in the vehicle-injected 5XFAD mice (Figure 2B). These results suggested that activation of the GHS-R1a might have anti-amyloid activity in the brains of patients with AD. 

### 2.3. The Ghrelin Agonist Significantly Attenuated Neurodegeneration in the Neocortex of 5XFAD Mice

**Figure 3:**
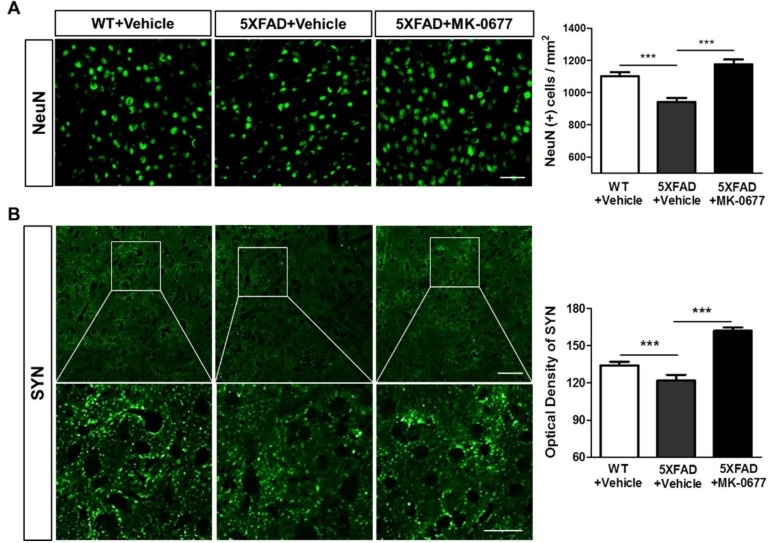
MK-0677-treated 5XFAD mice exhibited a significant reduction in neurodegeneration compared with the vehicle group. Immunofluorescent staining was performed to detect the markers of neuronal cells (NeuN) and pre-synaptic terminals (SYN) in layer V of the frontal cortex of wild-type (*n* = 8) and 5XFAD mice. MK-0677 significantly ameliorated the reduction of the number of NeuN (+) cells (**A**) and optical density of SYN (+) area (**B**) in 5XFAD mice (*n* = 6), compared with vehicle-treated 5XFAD mice (*n* = 7). *** *p* < 0.001 indicates significant differences between the groups. Scale bars are 50 μm in the upper panel and 25 μm in the lower panel.

We examined whether the anti-amyloid actions of MK-0677 affected the neuronal and synaptic loss in the AD brain. To visualize neuronal death and synaptic loss induced by Aβ, we immunohistochemically stained brain tissues with neuronal nuclear antigen (NeuN) and synaptophysin (SYN) The quantitative analysis showed that the number of NeuN-positive cells and SYN immunoreactivity were significantly decreased in the neocortex of vehicle-treated 5XFAD mice compared with the vehicle-treated wild-type mice (Figure 3). In contrast, the number of NeuN-positive cells per area (Figure 3A) and optical density of SYN (Figure 3B) were significantly higher in the MK-0677-administered 5XFAD mice compared with the vehicle-injected 5XFAD mice. These results demonstrated that neuronal and synaptic loss in the brains of 5XFAD mice was ameliorated by treatment with MK-0677.

### 2.4. Ghrelin Agonist Significantly Inhibited Neuroinflammation in the Deep Cortical Layers of 5XFAD Mice

**Figure 4:**
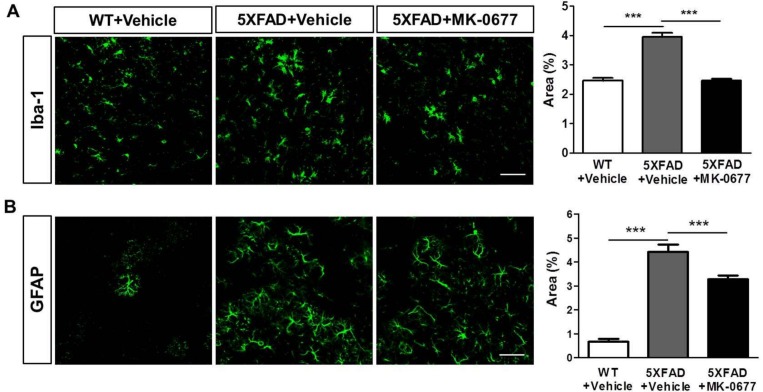
MK-0677-treated 5XFAD mice exhibited significant inhibition of neuroinflammation compared with vehicle-administered mice. Immunofluorescent staining was performed to detect the markers of microglia (Iba-1) and astrocyte (GFAP) in layer V of the frontal cortex of wild-type (*n* = 8) and 5XFAD mice. MK-0677 significantly reduced the Iba-1 (+) area (**A**) and GFAP (+) area (**B**) in 5XFAD mice (*n* = 6), compared with vehicle-treated 5XFAD mice (*n* = 7). *** *p* < 0.001 indicates significant differences between the groups. Scale bar = 50 μm.

To examine the effects of MK-0677 on neuroinflammation induced by Aβ, we performed immunohistochemical staining for the microglia marker ionized calcium-binding adaptor molecule 1 (Iba-1) and astrocyte marker glial fibrillary acidic protein (GFAP). The quantitative analysis showed that the percentages of the Iba-1- and GFAP-stained areas were markedly increased in the vehicle-administered 5XFAD mice compared with the vehicle-treated wild-type littermate mice. However, the MK-0677-treated 5XFAD mice showed a significant decrease in the Iba-1-positive (Figure 4A) and GFAP-positive (Figure 4B) areas compared with those in the vehicle-treated 5XFAD mice. These results suggested that MK-0677 treatment alleviates the neuroinflammation induced by Aβ in the AD brain.

### 2.5. Ghrelin Agonist Significantly Increased Phosphorylation of CREB in Dentate Gyrus of the Hippocampus of 5XFAD Mice

**Figure 5:**
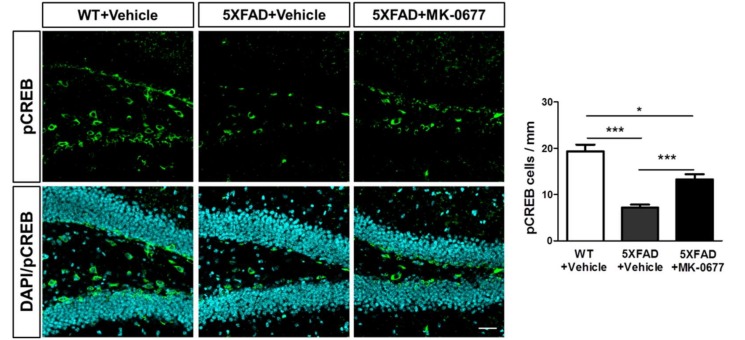
MK-0677 treatment significantly improved the reduced phosphorylation of CREB in 5XFAD mice. Immunofluorescent staining was performed to detect the phosphorylation form of CREB (pCREB) in dentate gyrus of the hippocampus of wild-type (*n* = 8) and 5XFAD mice. MK-0677-treated 5XFAD mice (*n* = 6) showed significantly increased pCREB, compared with vehicle-treated 5XFAD mice (*n* = 7). * *p* < 0.05 and *** *p* < 0.001 indicate significant differences between the groups. Scale bar = 50 μm. CREB = cyclic adenosine monophosphate (cAMP) response element binding protein.

To examine the effects of MK-0677 on the phosphorylation of CREB in dentate gyrus of the hippocampus, we performed immunohistochemical staining for the pCREB. The quantitative analysis showed that pCREB-immunostained cells per length of inner rim, defined as boundaries of hilus and granule layers, were markedly increased in the MK-0677-administered 5XFAD mice compared with the vehicle-treated 5XFAD mice (Figure 5). These result suggested that MK-0677 treatment inhibited the decrement of pCREB in dentate gyrus of the hippocampus in 5XFAD mice.

## 3. Discussion

Because the evidence for a correlation between the ghrelin system and neural function is growing, the roles of ghrelin in neurodegenerative and cognitive impairment diseases have been intensively investigated. Moreover, the effects of ghrelin on the pathological changes resulting from Aβ-induced pathogenesis might be used as a possible strategy for AD treatment. However, the previous randomized clinical trial reported that MK-0677 had no effects on cognitive function in patients with AD did not assess any pathologic changes, including Aβ levels [49]. Moreover, the effects of MK-0677 on Aβ accumulation in studies of ghrelin agonists and antagonists have been conflicting [46,47,48], which prompted us to examine if a ghrelin agonist reduced Aβ accumulation and Aβ-induced pathogenesis in AD animals. In the present study, we examined the effects of MK-0677 on AD pathogenesis in 5XFAD mice. The thioflavin-S staining and immunohistochemical results revealed that MK-0677 reduced Aβ accumulation, neurodegeneration, and neuroinflammation in the 5XFAD mice, which suggested that MK-0677 ameliorated Aβ accumulation, as well as Aβ-induced pathogenesis.

The therapeutic roles of exogenous GHS-R1a agonists in disorders, such as malnutrition, growth hormone deficiency, gastrointestinal hypomotility, and protein-energy wasting, are well known [53,54,55,56]. Despite the finding that a ghrelin agonist improved cognitive function in AD patients and animal models, the specific effects on Aβ accumulation varied [47,48,49]. We found that the intraperitoneal administration of MK-0677 significantly reduced Aβ accumulation in the frontal cortex in three-month-old 5XFAD mice. The deposition of Aβ started in the 5XFAD mice at two months and markedly increased at four months [57]. Moreover, several studies reported that the significant memory impairment was shown in the 5XFAD mice at 6 months of age [58,59,60,61,62].

Considering that Aβ deposits in the neocortex represent the first of the five phases of Aβ deposition and the patients with AD in the early phase of Aβ deposition do not exhibit dementia [63], our data indicated that the administration of a ghrelin agonist in the early stage of AD could alleviate Aβ accumulation before the onset of AD-related symptoms. The results of pCREB immunostaining showed that MK-0677 inhibited the decreased pCREB levels in dentate gyrus of the hippocampus and were corroborated by a previous study reporting the up-regulation of pCREB induced by increased ghrelin levels in the hippocampus [64]. It has been reported that hippocampal pCREB levels were down-regulated in AD patients or animal models of AD [65,66]. Accordingly, the inhibition of decreased pCREB in the hippocampus induced by MK-0677 might indirectly implicate that MK-0677 might conserve cognitive functions against Aβ-induced toxicity. However, these interpretations should be treated with caution because previous studies of ghrelin agonists in AD models and treatment have reported inconsistent findings of the effects of ghrelin agonists on Aβ accumulation [46,47]. Notably, the two studies had a difference in the diet of the animals: free diet vs. high-glycemic index diet. One possible explanation is that the altered glucose homeostasis induced by the high glycemic index diet influenced Aβ formation and that the ameliorating effects of ghrelin on glucose homeostasis were not sufficient to reduce the Aβ formation. Furthermore, results from a randomized clinical trial that showed MK-0677 had no cognitive improving effects in AD patients [49] made it hard to interpret our data. This disparity could be explained by the differences of AD stage of subjects between studies. The previous study included AD patients with mild to moderate dementia, whereas ours were mice with a very early stage of AD. Thus, these different results from studies might implicate that MK-0677 may be effective, at least, in an early phase of AD. Another possible limitation could be raised by studies supporting the roles of soluble Aβ oligomers on neurotoxicity [67]. Further investigations are needed to examine the effect of the ghrelin agonist on Aβ accumulation in models of altered glucose homeostasis and soluble Aβ oligomers, as well as behavioral tests for cognitive functions in AD animal models.

We next examined the effects of MK-0677 on neurodegeneration in the neocortex. NeuN and SYN, which are markers of neuronal and synaptic loss, respectively, were examined. The immunohistochemical results suggested that both neuronal/synaptic losses were reduced by the administration of the ghrelin agonist. In accordance with the neurodegenerative pathology in AD, Aβ accumulation is known to play key roles in that pathology [68]. The Aβ accumulation and accompanying neuronal loss were corroborated in a previous study that used the same transgenic mice as our study did [69]. Therefore, these results strongly suggested that treatment with the ghrelin agonist would sufficiently reduce neurodegeneration by inhibiting Aβ accumulation.

The results of the immunohistochemistry for Iba-1 and GFAP indicated that both microgliosis and astrogliosis were decreased in the deep layers of the neocortices by ghrelin treatment. These results were consistent with previous results that indicated that ghrelin suppressed gliosis in the CNS. The intraperitoneal injections of ghrelin inhibited microgliosis and the expression of inflammatory cytokines and nitric oxide synthase in the brain of an animal model of MPTP-induced Parkinson’s disease, and similar effects were found in 1-methyl-4-phenylpyridinium (MPP+)-treated cultured mesencephalic cells [70]. Ghrelin inhibits reactive oxygen species and inflammatory signaling proteins in microglial cells activated by lipopolysaccharide [71]. In addition, ghrelin decreases mRNA levels of tumor necrosis factor-α in primary cultured hypothalamic astrocytes [72]. Furthermore, in studies of the effects of the ghrelin agonist on neuroinflammation in AD model mice, the ghrelin agonist LY444711 subdued microglial activation [46]. However, the effects of the ghrelin agonist on gliosis in AD pathogenesis should be further examined because its effects could differ in AD patients with other conditions, such as an aberrant glucose metabolism [47].

Besides the beneficial effects of ghrelin on Aβ and Aβ-induced pathogenesis, ghrelin demonstrates orexigenic properties [14,73]. Ghrelin increases feeding by stimulating neuropeptide Y in the arcuate nucleus, as well as counteracted leptin [26,74]. We demonstrated that the administration of 3 mg/kg of MK-0677 resulted in an 11.6% increase in food intake and 64% weight gain in the C57BL/6 mice compared with the vehicle group. These findings were consistent with those of previous studies which reported that MK-0677 increased food intake and body weight [52,75,76]. Interestingly, MK-0677 did not increase the cumulative food intake or changes in body weight in the 5XFAD mice. Although the mechanisms underlying the lack of effects of ghrelin on food intake and body weight were not clear, it can be assumed that different species and strains may affect the results. Regardless of the specific mechanism, these results indicate that MK-0677 had beneficial effects on Aβ accumulation and Aβ-induced pathogenesis without changing appetite. Another possible explanation is suggested by the evidence, proposing that alterations of signaling pathways, such as the adenosine monophosphate (AMP)-activated protein kinase pathway, impair energy homeostasis in AD [77]. Thus, additional studies are needed to identify the effects of ghrelin on signaling proteins involving metabolism.

Hyperphosphorylation of tau and the resulting neurofibrillary tangles (NFTs) is another hallmarker of AD [78]. The Aβ-induced tau-pathology has been demonstrated by several studies, while its mechanism has not been fully elucidated [79,80]. It has been reported that only pS396 levels were increased in two-month-old 5XFAD mice [81]. However, despite the detection of abnormal tau phosphorylation in the brain, neurofibrillary tangles, the aggregates of hyperphosphorylated tau, are not observed as with other Aβ-related mutant mice (APP and APP/PS1) [69]. In addition, according to the results from a study using 5XFAD mice at three, six, and nine months of age, endogenous tau did not show age-related differences, while Aβ-related factors showed significant changes [82].

Concerning the signaling molecules involved in the abnormal phosphorylation of tau such as ghrelin treatment glycogen synthase kinase 3 beta (GSK-3), c-Jun N-terminal kinase (JNK), and cyclin-dependent kinases 5 (CDK5) [83], ghrelin treatment inactivated GSK-3β in spinal cord motoneurons [84]. In addition, treatment of ghrelin decreased the phosphorylation of c-Jun N-terminal kinase (JNK) in stimulated macrophages [85,86], hepatocytes [87], and hippocampal neurons [88]. Furthermore, although the relationship between ghrelin and CDK5 is not known, treatment of ghrelin increased the protein expression of CDK2 and Cyclin A in hippocampal neural stem cells [89]. Therefore, although tauopathy such as NFTs is inevident in 5XFAD, further studies are needed to determine the effect of ghrelin agonist MK-0677 on the phosphorylation of tau using other AD model mice.

An agonist needs its receptor for its action. GHS-R1a and GHSR-1 mRNA are expressed in various regions of the brain [90,91]. In patients with AD, even though GHS-R1a expression was decreased, a certain level of GHS-R1a was expressed [20]. Thus, the administration of exogenous ghrelin agonist decreased Aβ accumulation and improved Aβ-induced pathogenesis by stimulating GHS-R1a in the brain.

In the present study, the ghrelin agonist MK-0677 showed high potential for decreasing the burden of amyloid plaques. To solidify this result, studies on the precise molecular mechanisms of the Aβ-lowering effects of MK-0677 are essential. In other words, additional studies are needed to investigate the effects of MK-0677 both on amyloid precursor protein processing through the modulation of α-, β-, and γ-secretase, and the expression or activity of Aβ-degrading enzymes, such as neprilysin and insulin-degrading enzymes.

In conclusion, we demonstrated for the first time that the ghrelin agonist MK-0677 suppressed Aβ pathology and Aβ-mediated pathology in vivo. Our results suggest that MK-0677, at least, may be a promising therapeutic agent for the early phase of AD and that activation of the ghrelin receptor can be a therapeutic target for the treatment of AD.

## 4. Materials and Methods

### 4.1. Animals and Drug Treatment

Three-month-old male transgenic mice with five familial AD mutations (5XFAD) and B6JSLF1 mice were purchased from The Jackson Laboratory (Bar Harbor, ME, USA). The C57BL/6 mice were obtained from Korean Animal Technology (Koatech, Pyeongtaek-si, Gyeonggi-do, Korea). The 5XFAD mice had mutations in the amyloid precursor protein (*APP*) gene (Swe^K670N,M671L^, Lon^V717I^, and Flo^I716V^) and presenilin 1 (*PSEN1*) gene (M146L and L286V) regulated by the Thy1 promoter [69]. The number of mice per group was five for the C57BL/6 mice and six to eight for the 5XFAD and wild-type littermates. All mice were given access to food ad libitum and maintained under a 12-h light/dark cycle. The experimental procedures were designed in accordance with the National Institutes of Health guide for the care and use of laboratory animals [92] and performed under supervision of the Institutional Animal Care and Use Guidelines of Konyang University (project code:P-15-21-A-01; 30 November 2015).

Inasmuch as 5XFAD mice exhibit detectable Aβ deposits at two months of age [57,69], MK-0677 (Bio-Techne Corporation, Minneapolis, MN, USA) or a vehicle was administered at the age of three months. MK-0677 was dissolved in saline and administered by intraperitoneal injections at a dose of 5 mg/kg daily for three weeks. The vehicle groups were injected with saline in parallel. The animals were sacrificed one day after the last administration. Food intake and body weight were measured before administration during the period of treatment.

### 4.2. Brain Tissue Preparation

The animals were anesthetized and transcardially perfused with 0.05 M phosphate-buffered saline (PBS) and then fixed with ice-cold 4% paraformaldehyde in 0.1 M phosphate buffer (PB). The brain tissue was removed, postfixed in 0.1 M PB containing 4% paraformaldehyde (Sigma-Aldrich Corporation, St. Louis, MO, USA) for 20 h at 4 °C, and then immersed in 30% sucrose in 0.05 M PBS for three days at 4 °C for cryoprotection. The samples were sliced into 30-μm serial coronal sections on a cryostat (Leica Biosystems Nussloch GmbH, Nussloch, Germany). The serial coronal sections were stored in storing solution (25% ethylene glycol and 25% glycerol in 0.05 M PB) at 4 °C until use for the histological analysis.

### 4.3. Thioflavin-S Staining

Six brain sections at 210–240-μm intervals were extracted from each mouse from the region between −2.6 mm and −4.3 mm to the bregma with reference to Paxinos and Franklin’s the Mouse Brain in Stereotaxic Coordinates [93]. To label Aβ, the stored brain sections were washed three times for five minutes in PBS. The sections were incubated in filtered thioflavin-S solution (1%; Sigma-Aldrich Corporation, St. Louis, MO, USA) for 15 minutes and washed in 80% and 70% ethanol for one minute each. After washing three times for five minutes each in PBS, the sections were mounted on ProbeOn™ Plus Microscope Slides (Thermo Fisher Scientific Inc., Waltham, MA, USA) and coverslipped with the Fluoroshield™ with DAPI (Sigma-Aldrich Corporation, St. Louis, MO, USA).

### 4.4. Immunofluorescence Labeling

Six brain sections at 210–240-μm intervals were extracted from each mouse from the region between −2.6 mm and −4.3 mm to the bregma with reference to Paxinos and Franklin’s the Mouse Brain in Stereotaxic Coordinates [93]. To visualize the immunoreactivity of Aβ, NeuN, Iba-1, SYN, GFAP, and pCREB, free-floating sections were incubated overnight at 4 °C with the mouse anti-4G8 antibody (1:2000; BioLegend, San Diego, CA, USA), mouse anti-NeuN antibody (1:100; Merck KGaA, Darmstadt, Germany), goat anti-Iba1 antibody (1:500; Abcam plc, Cambridge, UK), mouse anti-SYN antibody (1:500; Sigma-Aldrich Corporation, St. Louis, MO, USA), rat anti-GFAP (1:200; Thermo Fisher Scientific Inc., Waltham, MA, USA), or mouse anti-pCREB antibody (1:1000; MERCK, Kenilworth, NJ, USA). After washing three times for five minutes in PBS, the sections were incubated with the goat Alexa 488-conjugated anti-mouse IgG (1:200; Thermo Fisher Scientific Inc., Waltham, MA, USA) or donkey Alexa 594-conjugated anti-rabbit IgG (1:200; Thermo Fisher Scientific Inc., Waltham, MA, USA) for 1 h at room temperature. The tissue sections were mounted on ProbeOn™ Plus Microscope Slides (Thermo Fisher Scientific Inc., Waltham, MA, USA) and coverslipped with Fluoroshield™ with DAPI (Sigma-Aldrich Corporation, St. Louis, MO, USA).

### 4.5. Image Acquisition and Quantification

The labeled tissues were imaged and analyzed with a Zeiss LSM 700 Meta confocal microscope (Carl Zeiss AG, Oberkochen, Germany). We examined amyloid plaques, neurodegeneration and neuroinflammation in the deep cortical layers of the frontal cortex, and pCREB in dentate gyrus of the hippocampus. To quantify the fraction of the stained areas and immunoreactivity, we analyzed the images using ImageJ software (National Institutes of Health, Bethesda, MD, USA), as described in our previous study [94].

### 4.6. Statistical Analysis

The Graph Pad Prism 5 software (GraphPad Software, La Jolla, CA, USA) was used for data representation and statistical analysis. All results are shown as means ± standard error of the mean. The differences between the treatments were analyzed by one-way analysis of variance, which was followed by Fisher’s least significant differences post hoc test or an independent *t*-test. *p* values less than 0.05 were considered statistically significant.

## 5. Conclusions

The results of thioflavin-S staining and immunohistochemical staining results showed that the administration of MK-0677, a ghrelin agonist, attenuated Aβ deposition and Aβ-mediated pathologies in the neocortex of three-month-old 5XFAD mice. Moreover, MK-0677 inhibited the decreased pCREB levels in dentate gyrus of the hippocampus. Taken together, we suggest that MK-0677 might be have potential for the treatment of early phase AD.
